# A qualitative interview study exploring pregnant women’s and health professionals’ attitudes to external cephalic version

**DOI:** 10.1186/1471-2393-13-4

**Published:** 2013-01-16

**Authors:** Rebecca Say, Richard Thomson, Stephen Robson, Catherine Exley

**Affiliations:** 1 Institute of Health and Society, Baddiley - Clark Building, Richardson Road, Newcastle Upon Tyne, NE2 4AX, UK; 2 Institute of Cellular Medicine, 4th Floor, William Leech Building, Medical School, Framlington Place, Newcastle University, NE2 4HH, UK

**Keywords:** Pregnancy, Breech presentation, External cephalic version, Mode of delivery, Attitudes, Shared decision making

## Abstract

**Background:**

Women who have a breech presentation at term have to decide whether to attempt external cephalic version (ECV) and how they want to give birth if the baby remains breech, either by planned caesarean section (CS) or vaginal breech birth. The aim of this study was to explore the attitudes of women with a breech presentation and health professionals who manage breech presentation to ECV.

**Methods:**

We carried out semi-structured interviews with pregnant women with a breech presentation (n=11) and health professionals who manage breech presentation (n=11) recruited from two hospitals in North East England. We used purposive sampling to include women who chose ECV and women who chose planned CS. We analysed data using thematic analysis, comparing between individuals and seeking out disconfirming cases.

**Results:**

Four main themes emerged from the data collected during interviews with pregnant women with a breech presentation: ECV as a means of enabling natural birth; concerns about ECV; lay and professional accounts of ECV; and breech presentation as a means of choosing planned CS. Some women’s attitudes to ECV were affected by their preferences for how to give birth. Other women chose CS because ECV was not acceptable to them. Two main themes emerged from the interview data about health professionals’ attitudes towards ECV: directive counselling and attitudes towards lay beliefs about ECV and breech presentation.

**Conclusions:**

Women had a range of attitudes to ECV informed by their preferences for how to give birth; the acceptability of ECV to them; and lay accounts of ECV, which were frequently negative. Most professionals described having a preference for ECV and reported directively counselling women to choose it. Some professionals were dismissive of lay beliefs about ECV. Some key challenges for shared decision making about breech presentation were identified: health professionals counselling women directively about ECV and the differences between evidence-based information about ECV and lay beliefs. To address these challenges a number of approaches will be required.

## Background

Breech presentation affects 3-4% of singleton term pregnancies [1]. Women who have a breech presentation have two key decisions to make: whether to attempt external cephalic version (ECV) and how they want to give birth if their baby remains breech, either by planned caesarean section (CS) or attempted vaginal breech birth. A Cochrane review demonstrated that ECV was associated with a reduction in non-cephalic birth and CS; and that ECV was not associated with increased perinatal morbidity or mortality [1]. ECV has also been shown to be cost-effective [2]. Despite the evidence in favour of attempting ECV, it may not always be offered and reported uptake varies from 24% to 74% [3,4]. A Cochrane review found planned CS for breech presentation was associated with a lower risk of perinatal or neonatal death and short term neonatal morbidity compared with vaginal breech delivery, but there was an increased risk of short-term complications for mothers, and insufficient evidence to evaluate the implications of planned CS for future pregnancies [5].

Engaging pregnant women in shared decision making (SDM) has become an important part of obstetric care [6]. SDM is a process in which patients and clinicians collaborate together to make decisions about health care. It involves health professionals and patients communicating together so that clinicians can: share evidence-based information about options with patients; support patients in deliberating about the options; facilitate patients developing informed preferences for treatment (or screening) based on their values and health goals; and help implement the decisions made [7]. Internationally, SDM has become widely advocated as the ideal model of decision-making in many clinical situations, particularly when there is no overall best choice, but it may be challenging for some clinicians [7,8].

For clinicians to support women in SDM about breech presentation they need to communicate information about treatment options, and understand women’s attitudes to ECV and mode of delivery for breech presentation. Education about treatment options can be informed by high quality, if complex, information in the form of Cochrane reviews and clinical guidelines. However, at present there is little research evidence about women’s and health professionals’ attitudes to ECV.

Two qualitative studies explored the attitudes of women towards breech presentation [9,10]. Guittier et al. explored Swiss women’s experiences of making decisions about mode of delivery for breech presentation. They reported that participants were highly motivated to have their babies turned by attempting ECV or complementary therapies [10]. Founds reported that some women in rural Jamaica were anxious about the diagnosis of breech presentation perceiving dangers for both mother and baby from vaginal breech birth [9]. However, neither study explored women’s attitudes towards ECV.

In previous cross-sectional surveys, respondents had varied attitudes towards ECV. Reasons given for choosing ECV included desire to avoid CS and to deliver naturally and doctor’s advice [11,12]. Positive features of ECV respondents reported included having an additional ultrasound examination and the fetal monitoring during the procedure [13]. Reasons given for declining ECV included doctor’s advice; concerns about safety, including cord entanglement and abruption; failure rate; fear of reversion; pain; a perception that ECV is unnatural; inability to guarantee vaginal delivery even if ECV is successful; and a preference for CS [4,11,12]. These cross-sectional surveys were limited in their ability to collect in-depth data about women’s attitudes. Furthermore, two of the studies surveyed pregnant women who did not have personal experience of breech presentation, so these women’s responses were theoretical [4,12].

The aim of this study was, therefore, to explore in-depth the attitudes of both women with a breech presentation and health professionals who manage breech presentation towards ECV. This was part of an ongoing investigation into the needs of pregnant women and health professionals to support decision making about breech presentation.

## Methods

Ethical approval for this study was given by Sunderland Research Ethics Committee (ref 08/H0904/89). We recruited pregnant women and health care professionals involved in managing breech presentation in two hospitals in the North East of England (one teaching hospital with 6400 deliveries per year and one district general hospital with 3200 deliveries per year) between May-July 2009. We used purposive sampling to include both women who had chosen ECV and women who had chosen planned CS. We also included both primiparous and multiparous women.

Pregnant women with a breech presentation were initially approached by a member of their clinical team; provided with a participant information sheet; and asked if they would consider providing their contact details to allow us to contact them after they had had time to consider participation. They were given a minimum of 48 hours to consider participation in the study before being contacted to arrange an interview at a time and place which was convenient for them (either at home or in a private location in the hospital). We approached health professionals directly with an information sheet about the study and re-contacted them, following time to consider participation, to arrange a suitable time and place for an interview (either in their office or another private location in the hospital). We obtained written informed consent prior to the interviews and participants were informed their data would be kept confidential and that identifiers would be removed from the data to enable anonymity.

We used semi-structured interviews (which lasted up to 45 minutes) for data collection to allow detailed exploration of participants’ experiences of decision making about ECV and breech presentation. We developed interview schedules by critically reviewing the literature and from our experience of developing decision support in other clinical situations. We used open ended questions to avoid limiting discussion through prior categorisation or by structuring interviews around the researcher’s ideas and assumptions. The topic guide is included as Additional file 1.

We analysed interview transcripts systematically using thematic analysis employed in five stages: familiarisation with the data through reading and rereading the transcripts for recurrent themes; coding of the transcripts based on the objectives of the study and emergent themes; comparing codes between interviews and re-coding if necessary; developing a conceptual framework grouping themes together into broader categories to facilitate interpretation; and summarising and synthesising data into charts that used representative quotes to demonstrate themes.

We considered validity by using interview schedules to provide a standardised approach (although flexible in order to ensure responsiveness); being reflexive throughout; and by undertaking rigorous, systematic analysis of the transcripts, comparing between individuals, seeking out alternative explanations and disconfirming cases. When reporting results, we have included a selection of quotes from the interview data to allow readers to scrutinise the analytic process and assess validity. We selected quotations because they reflected or summarised accurately the experiences of a number of participants or because they were examples of disconfirming cases and showed a different perspective or experience.

## Results

### Participants

Of 14 pregnant women contacted, 13 agreed to participate. Two women later withdrew from the study prior to being interviewed (no reasons given). Seven women were nulliparous and four multiparous. Four women chose to have a planned CS and seven chose to attempt ECV. Three women had undergone ECV by the time they were interviewed. Two of these were successful and one unsuccessful. One multiparous participant who chose ECV experienced spontaneous version before the planned ECV. None of the participants chose to have a vaginal breech birth. Of 12 health professionals approached, 11 agreed to participate (nine consultant obstetricians; one final-year specialist registrar and one midwife who performed ECVs).

### Women’s attitudes towards ECV

Four main themes emerged from the data: ECV as a means of enabling natural birth; concerns about ECV; lay and professional accounts of ECV; and breech presentation as a means of choosing planned CS.

### ECV as a means of enabling natural birth

Women who chose to attempt ECV perceived it as a way of enabling ‘natural’ birth and avoiding CS. Many participants expressed a strong preference for vaginal birth.

I really want to experience giving birth and being in labour. This is our first baby and I’m not frightened of it, I’m not terrified of being in labour and I really want to experience that as much as possible and, fingers crossed, have a natural birth. **Woman 8, nulliparous and chose ECV**

Many women saw CS as major surgery, and a last resort, and preferred a vaginal birth because they perceived it as: ‘natural’; safer for their baby than CS; likely to enable a faster recovery after the birth; and it was an achievement which would enhance their self-esteem.

I’ve never had an operation before so it’s quite nerve-racking really to think about that and just quite scared of it really I suppose…The recovery and not being able to drive and get out and about and stuff as much as, so everything really I just didn’t want one. **Woman 7, nulliparous and chose ECV**

Yes it’s a very nice idea to have yourself cut open and in an hour later your baby put in your arms but it’s a fact that you know you are suffering still, 4–6 weeks later… our bodies are built to give birth vaginally I believe so let nature take its course and do it that way. **Woman 3, multiparous and chose ECV but experienced spontaneous version**

For some women, the diagnosis of breech presentation represented a shift from ‘normal’ to ‘abnormal’ pregnancy. Several women described having anticipated a low risk birth, such as water birth, prior to the diagnosis and that having a breech baby had required them to reconsider their plans for birth.

I think initially I was a bit upset because at that point it was the first time I thought about the birth. One of my friends had a pool birth and I thought that would be quite nice, I might ask about that and I literally asked my midwife about that then the following week when she was like, “well she’s breech”. **Woman 2, nulliparous and chose planned CS**

For some women ECV represented a way to transition back to ‘normal’.

Obviously he’s head down and I can do it normally now. **Woman 10, nulliparous and chose ECV**

No participants preferred vaginal breech birth to ECV or planned to have a vaginal birth if their ECV was unsuccessful. Vaginal breech birth was seen as risky for babies and painful.

I’ve got a friend who’s had a breech birth and she said it was the most painful thing she’d ever experienced… If she had the option she would always go for a caesarean. **Woman 10, nulliparous and chose ECV**

### Concerns about ECV

Participants described a number of concerns and worries about ECV including pain during the procedure; believing ECV was unnatural, the risks of ECV; and the 50% quoted success rate of ECV. Women commonly worried that having an ECV would be painful. For some women this fear of possible pain contributed to their decision to have a planned CS. Some women reflected that they had found routine antenatal abdominal examinations uncomfortable so worried that they would be unable to tolerate an ECV. Others described that they already felt tight across their abdomen and were concerned that there was not enough space for the baby to turn.

I’m slightly worried because my stomach seems very tight. There doesn’t seem to be a lot of space, so obviously you just think that it’s just going to hurt you know and obviously you don’t really want the pain. **Woman 4, multiparous and chose ECV**

However, all the women who had undergone ECV prior to being interviewed reported the level of discomfort had not been as bad as they had expected.

I was a bit scared to have it done at first actually because, obviously people, friends and things who haven’t had it done all were saying it’ll hurt. But to actually have it done, it didn’t, I was expecting some kind of pain, but it didn’t hurt as much as what I thought. So, like she said like if I had to have it done again, yeah, I would have it done again. **Woman 9, multiparous and chose ECV**

Several women saw ECV as unnatural and it was commonly referred to as a ‘manipulation’. Some participants opted for CS because they perceived their baby had ‘chosen’ to be breech so it would be unnatural to attempt to turn the baby. Some women had also assumed ECV would involve a vaginal examination. Thus, ECV was seen by some as an invasive procedure.

I thought it would be internal and I didn’t know until last week that it’s actually a manipulation of your stomach… I personally feel as though the baby’s been up here forever…and I just thought…if it wants to be there it wants to be there. **Woman 5, nulliparous and chose planned caesarean section**

Some participants expressed specific concerns about the risks of ECV. These included fetal distress; bleeding (and for some participants the need for additional anti-D); the possibility of needing an emergency CS; and the risk of membrane rupture. Some participants expressed particular anxiety about rare, but serious, complications such as cord entanglement and placental abruption. For some women these risks put them off ECV.

From what I’ve read medically, I know there’s a tiny, tiny chance it could bring on labour, a tiny chance the baby could get distressed, again I know they are tiny chances but I don’t like those options. **Woman 2, nulliparous and chose planned CS**

Other women appeared to accept the risks as they perceived them as rare and were reassured by the monitoring they would receive (or had received) during an ECV.

I didn’t realise that everything would be monitored as closely as it was. I think that was something that could have been missing from the information. To explain that you are fully monitored throughout the process and what the monitoring of the process actually was. So that was nice to see the baby on the screen and to hear what was happening at the same time. **Woman 11, nulliparous and chose ECV**

I knew that if baby became distressed I’d be straight to theatre and sorted out straight away. So that’s fine. **Woman 3, multiparous and chose ECV but experienced spontaneous version**

For some women, the success rate was another factor which contributed to their decision not to have an ECV, as they perceived 50% as too low.

If it had have been a high percentage then maybe I would have but I think at that point I had already made me mind up…to have a c-section, I just personally didn’t see the point. Just didn’t think it would work. **Woman 5 nulliparous and chose planned caesarean section**

Women were also aware that there was a risk that the baby could revert to breech presentation after a successful ECV. Some women had friends or relatives who had experienced this and this contributed to their decision to not attempt ECV.

I’ve spoken to a few that turned and then it’s flipped back round…I think if there wasn’t those people out there that had those experiences then it might be a different thing. **Woman 2, nulliparous and chose planned CS**

### Accounts of ECV

Most participants had spoken to friends or relatives about ECV and knew someone who had had a breech baby previously. Many women described accounts of ECV they had been given by their friends and family (some of whom had personal experience of ECV and some of whom did not). ECV had often been portrayed negatively by other women as painful, unsuccessful or dangerous. Some women had been put off ECV by these lay accounts.

I think the reason we chose to have a planned caesarean was we’d spoken to quite a few people who’d tried to have babies turned and they all said that it was quite painful and baby got a bit distressed. **Woman 1, nulliparous and chose planned caesarean section**

Other participants were more ambivalent to negative lay accounts and dismissed others’ negative experiences of ECV, believing themselves to be different in some way. For example, they believed that their baby might be in a more favourable position.

She just said it was very uncomfortable and it obviously didn’t work for her but her baby was folded in half in a way. The bum was right in the pelvis… I think my baby’s in a slightly different position so hopefully it might be a bit more successful. **Woman 4, multiparous and chose ECV**

In contrast to the lay accounts women described, participants reported that most health professionals (including community midwives, hospital midwives and obstetricians) were positive about ECV and encouraged them to consider it.

It was the way that she explained it. She was friendly and calm and kind and didn’t expect us to have to have it but was enthusiastic about it which made it seem so much like it was an everyday thing. **Woman 10, nulliparous and chose ECV**

She just made me feel at ease because she said it can be not, it’s not painful, that’s the worry that it is going to be painful… and that just made me feel a lot better about it. So when (name of obstetrician) did come in I wasn’t as anxious as what I thought I was going to be. **Woman 11, nulliparous and chose ECV**

### Means of choosing CS

Some women chose not to have an ECV because they had a preference for planned CS, sometimes irrespective of the presentation of their baby. They explained that having a CS would enable them to avoid a painful labour, vaginal birth and perineal injury, and perceived that choosing a planned CS offered them control over their birth. Some women described how a CS was more convenient for them, so that they could make arrangements for their birth and any additional support that they would need. Some women explained that they chose a planned CS because they did not want to have an emergency CS during labour.

I’m one of those people that likes to know what’s going to happen… even the thought of knowing the date your baby’s coming with a caesarean, knowing you’re getting up that morning knowing you are going to have your baby that day, I can’t say that in itself hasn’t had an impact as well… you could go for the natural labour option and then end up being rushed in, emergency c-section I think’s probably would be my biggest scare. **Woman 2, nulliparous and chose planned CS**

Having a breech presentation facilitated negotiation with health professionals about planned CS. One woman, who had already expressed a preference for a planned CS due to a previous traumatic birth, found her obstetrician reluctant to agree until the diagnosis of breech presentation was made. This may not be so important when planned CS is available to women without a clinical indication.

Anyway, so that’s, a caesarean has been you know, something that I wanted from the very beginning. Whether I wished my baby to be breech, I don’t know. But anyway the baby is breech so I mean a caesarean is what I’m going to have to have anyway. **Woman 6, multiparous and chose caesarean section**

### Health professionals’ attitudes towards ECV

Two main themes emerged from the interview data about health professionals’ attitudes towards ECV; directive counselling and attitudes towards lay beliefs about ECV and breech presentation.

### Directive counselling

Health professionals were generally very positive about ECV. They perceived that it was a safe procedure, a good way to avoid CS and that women have nothing to lose by attempting it. Several professionals acknowledged a preference for ECV for the management of breech presentation and that they would try to persuade women to choose one.

I think we should be promoting ECV within the context of it being a safe procedure. **Health Professional 8**

One professional suggested clinicians should be up front with women and admit their own preference. Some participants described trying to influence women into making the decision that they felt was most appropriate.

I would certainly fight, I would try and direct her to having an ECV because I think it’s such a shame if it’s, you get to know whether you think it’s going to be easy or not and I think it would be such a shame to have a section needlessly if you think a baby would turn. **Health Professional 11**

Furthermore, participants were sometimes critical when women’s preferences did not match their own. For example, many participants perceived that some women welcome breech presentation as a justification for a planned CS which would otherwise not be sanctioned by a clinician. They suggested that these women do not want to be informed about other options and that they do not make an informed choice.

I do want them to listen and then if I feel that they still don’t make an informed decision really because they have this one direction view and they don’t look at the other things I tried to tell them, I do try to steer them sometimes. **Health Professional 6**

I think many think it’s their ticket to convenient caesarean section… I think there will be a proportion of women will not be interested in any form of information that you provide for them when they don’t have to take up the test because they can have the operation that provides them with a number of other consumerist goals that they wish to meet. **Health Professional 9**

### Attitudes towards lay beliefs

Participants consistently perceived that women’s preferences were based on misinformation in the community. Commonly held beliefs they reported hearing from women were that ECV was dangerous, an unpleasant experience and that vaginal breech birth was dangerous. They suggested that women over-estimate the risks of ECV because of these beliefs. They also suggested that pain from an ECV might be seen as unacceptable and contrasted that with the expected pain of delivery whether vaginal or abdominal.

It’s sub-culture and I think we as doctors and even the midwives sort of enter that arena very late in the process because access to the pregnancy sub-culture starts long before a woman’s ever even pregnant you know. If I were to, if you were to ask a whole bunch of young women who have not got babies and who are not pregnant what they thought about the breech births, I bet a lot of them know about it and they’ll have already decided that they’re not going to have a vaginal birth cos it was a bad thing and the baby’ll get damaged. **Health Professional 3**

I spoke to my third cousin twice removed and they said definitely don’t have that or they had it or my granny had it when she was pregnant and it was awful and some people come in with very fixed ideas. **Health Professional 11**

I think women over-estimate the risks of ECV and some women over-estimate the discomfort of ECV. **Health Professional 8**

They feel it’s possibly unnecessary pain compared to labour pain or labour pain or caesarean section pain. **Health Professional 10**

Professionals believed that women have unrealistic expectations of labour in general and particularly of CS, some of which may have been influenced by the presentation of celebrity pregnancies in the media. They also acknowledged that the experience of previous deliveries might affect women’s preferences for ECV, and that women valued the safety of their baby highly. Some participants were concerned that some women might be put off ECV because the procedure is unfamiliar and difficult to imagine, in contrast to CS, and that some women may not be able to understand the risks of CS particularly if presented at a population level. Participants did not acknowledge that they themselves may not communicate effectively about risks and benefits of the different options.

I think risks, previous community based rumour and speculation from family, from friends, from other healthcare… Well I think my feeling is the majority are more concerned about the risk to the unborn child rather than to themselves, and that appears to be the maternalistic approach to it. **Health Professional 4**

So I think most people’s problem with ECV is a failure on their part, quite understandably, to be able to visualise what on earth you are going to do … I don’t think that women are actually able …to put into perspective what the implications are of having a caesarean section, because they can’t really appreciate, some will, but the vast majority won’t appreciate how to translate population statistics into their own individual, into what it means to them as an individual. **Health Professional 9**

## Discussion

This is the first qualitative study to explore, compare and contrast women’s and health professionals’ attitudes towards ECV. The use of semi-structured interviews to collect data enabled an in-depth exploration of attitudes that was not possible in previous cross sectional surveys, and the findings contribute to the limited evidence base on women’s and clinicians’ experiences of and beliefs about breech presentation.

Four main themes emerged from the data from the interviews with women with a breech presentation. The first theme was ECV as means of enabling natural birth with many participants perceiving it as a way of achieving the vaginal birth they wanted. The second theme was women’s concerns about ECV, in particular concerns about pain, risks and likely success. The third theme was lay and professional accounts of ECV which were often in contrast. The final theme was breech presentation as a means of choosing planned CS. Some women declined ECV because they had a preference for planned CS to avoid a painful labour, vaginal birth and possible perineal injury and because they perceived that planning a CS gave them control over the birth. Two main themes emerged from the interview data about health professionals’ attitudes towards ECV; directive counselling and attitudes towards lay beliefs about ECV and breech presentation.

Consistent with previous quantitative research, women described varied attitudes to ECV. Some women’s decisions about ECV appeared to be strongly influenced by their attitudes towards vaginal birth and CS. Many participants had also discussed breech presentation and ECV with friends and relatives, and many of the lay accounts of ECV they described were discouraging to them. Both Guittier et al. and Founds also described that women sought information about mode of delivery for breech presentation from their social networks and that the accounts they received included negative information, focusing on the risks of breech presentation [9,10]. Guittier et al. found that women reported they had made a decision about mode of delivery before being given information or counselled at the hospital [10]. Similarly, in this study, women reported being put off ECV by friends and relatives who had told them ECV was painful; likely to be unsuccessful; that babies often subsequently reverted to breech presentation; and that babies become distressed.

In contrast to the negative lay accounts of ECV women in this study described, they reported that health professionals had been positive about ECV. In agreement with this, participating professionals generally reported having a preference for ECV and were open about their practice of trying to convince women to choose it. They were concerned about women making their decisions based on what they perceived as inaccurate lay beliefs and misconceptions about ECV. As experts, they positioned themselves as the most appropriate source of information for women.

These findings reveal some challenges for SDM about breech presentation. Firstly, SDM may not occur as many health professionals described a clear preference for attempting ECV and reported counselling women directively. Some professionals seemed to define the ‘right decision’ as the one which matched their own opinion and values rather than the woman’s. Decision quality has been defined as ‘the extent to which a decision reflects the considered preferences of a well-informed patient, and is implemented’ [14]. Thus if a woman understands the pros and cons of each option, but highly values the outcomes associated with planned CS (for example avoiding the discomfort of ECV), by making that choice she has made a high quality decision as she is informed and has selected the option which best matches her own health goals. This may be challenging for health professionals, particularly when women’s preferences do not match their own or if they believe women have been influenced by accounts of ECV in the community which they perceive to be inaccurate.

Furthermore, health policy, while rhetorically supporting patient choice (‘no decision about us without us’[15]) may include recommendations which potentially obstruct shared decision making. For example, the NHS Institute for Innovation and Improvement made recommendations to reduce England’s CS rate [16] and promoted directive counselling to achieve this. For example, they suggested that maternity units should aim for 80% uptake of vaginal birth after caesarean section (VBAC) by encouraging women to choose VBAC rather than a repeat CS. While they recommended that all eligible women should be offered ECV, they did not set a target for uptake. Nevertheless, increasing ECV uptake has been identified internationally as a potential way to reduce CS rates [17,18].

To overcome these challenges a variety of approaches may be needed. Education and training for health professionals to inform them about SDM and to help them develop relevant competences such as risk communication; methods of exploring women’s attitudes towards treatments; and ways to discuss lay beliefs without appearing dismissive [19]. Antenatal education could also prepare women for SDM, by informing them about what to expect, such as to expect the professional to ask them to consider what is important to them in making a particular decision. Clinicians (and politicians) should also be explicit about health policy which seeks to influence the choices women need to make in pregnancy.

Further support for decision making about breech presentation might also be helpful, such as a decision aid. Decision aids are interventions which can help people make choices about healthcare by providing information about treatment options –in this case evidence-based information about ECV, CS and vaginal breech delivery- and by clarifying personal values [20,21]. A decision aid consisting of a workbook and audio component has been developed for decision making about ECV by a group in Australia [22]. Results were promising, as women who used it had higher knowledge, lower decisional conflict, were more satisfied with the amount of information they had been given and were more likely to state that they intended to have an ECV [22]. There was no difference in anxiety; the proportion of women actually choosing ECV; or in the rate of CS [22]. Using a web-based decision aid may enable women to readily access high quality information whenever, and with whomever, they prefer and should help them clarify their own attitudes about options, in preparation for discussion with health professionals. Common misconceptions about ECV or the different methods of delivery could also be addressed, and women could be encouraged to discuss any conflicting lay accounts of ECV or delivery methods with their clinicians. We are currently developing such a web-based decision aid.

A limitation to this study was that, during interviews, RS was open about her role as a trainee obstetrician and also answered any clinical questions which arose (or referred women back to their clinical team if she could not answer the question). Thus, participants may have been affected by knowing the interviewer had prior knowledge and experience herself of managing breech presentation and by her role as a doctor. However, RS was not involved in the clinical care of any participants. Limitations to interviewing health professionals may have included difficulty in accessing their underlying beliefs due to them being experienced in presenting themselves in public [23]. All the professionals were known to RS which may also have affected the interactions. Findings of the study may not be generalisable to other settings and interpretive limitations such as over-complexity or reductionism are possible.

## Conclusions

Women in this study described varied attitudes towards ECV. Some women’s attitudes appeared to be affected by their prior preferences for how to give birth. Other women chose CS because ECV was not acceptable to them. Women’s decisions about ECV were often influenced by lay accounts of ECV which were frequently reported as negative. Most professionals described having a preference for ECV and reported trying to counsel women to choose it. They were also dismissive of lay beliefs about ECV. The study has identified some challenges for SDM about breech presentation including: health professionals counselling women directively about ECV and the differences between evidence-based information professionals’ perceive as important for decision making and lay beliefs which seem to be important to many women. To address these challenges a number of approaches will be required.

## Abbreviations

ECV: External cephalic version; CS: Caesarean section; SDM: Shared decision making.

## Competing interests

The authors declare that they have no competing interests.

## Authors’ contributions

All four authors conceived and designed the study, analysed and interpreted the data, revised the article and approved the final version. RS drafted the article and will act as guarantor.

## Authors’ information

Rebecca Say holds a Doctoral Research Fellowship from the National Institute of Health Research to undertake a PhD study ‘Engaging women in shared decision making about breech presentation at term: development, usability testing and a pilot trial of a patient decision aid and decision quality instrument’.

Richard Thomson leads the Decision Making and Organisation of Care research programme in the Institute of Health and Society, Newcastle University and co-leads a programme on implementation of SDM (MAGIC – Making Good Decisions in Collaboration) with Glyn Elwyn in Cardiff, supported by the Health Foundation.

Stephen Robson is Professor of Fetal Medicine at Newcastle University and is an Honorary Consultant Obstetrician in The Newcastle upon Tyne Hospitals NHS Foundation Trust and has a research interest in ECV.

Catherine Exley is Senior lecturer in Medical Sociology, Institute of Health and Society, Newcastle University.

## Pre-publication history

The pre-publication history for this paper can be accessed here:

http://www.biomedcentral.com/1471-2393/13/4/prepub

## Supplementary Material

Additional file 1 **Topic Guides. **Topic Guide for women with a breech presentation. Click here for file
