# Supplementary material for: A qualitative interview study exploring pregnant women’s and health professionals’ attitudes to external cephalic version
Source: BMC Pregnancy Childbirth. 2013 Jan 16;13:4. doi: 10.1186/1471-2393-13-4 (PMC3567941; doi:10.1186/1471-2393-13-4)
Supplement: Additional file 1 — Topic Guides. Topic Guide for women with a breech presentation. [file 1471-2393-13-4-S1.docx]

# Appendix 1: Topic Guides

## Topic Guide for women with a breech presentation

1. **Introduction of researcher and recap the purpose of the study.**
2. **Acknowledge that the interview will be confidential**
3. **Do you have any questions or concerns about the study?**
4. **Which of the following best describes how you would want to make a decision about breech presentation (using a show card with the options printed in large font)?**
   - I would prefer to make the final selection about whether I have an ECV or an elective Caesarean section.
   - I would prefer to make the final selection about whether I have an ECV or an elective Caesarean section after seriously considering my doctor's opinion.
   - I prefer that my doctor and I share responsibility for deciding about whether I have an ECV or an elective Caesarean section..
   - I prefer that my doctor makes the final decision about whether I have an ECV or an elective Caesarean section but seriously considers my opinion.
   - I prefer to leave all decisions regarding my treatment to my doctor.
5. **Do you know what options will be available to you if you have a breech baby?**

**If not known state that there is the option to have the baby turned (ECV) or the option to do nothing and decide about how you want your baby to be born (usually by Caesarean section).**

**What do you know about these different options?**

- - What are the risks and benefits of each method?
  - If pain is raised as a disadvantage of ECV ask how she would feel if it were available under a regional anaesthetic (explain usually a spinal anaesthetic so she would be awake but unable to feel pain).

1. **Do you know which option you prefer**
   - **Why do you prefer that option?**
2. **What kind of information was/ would be helpful in making your choice?**
3. **What do you think is important to women when making a choice about breech presentation?**
4. **How would you like to be given information?**
   - **What do you think about information given as**
5. leaflets
6. as a computer programme
7. on the internet
8. As a DVD
9. **How do you prefer information to be presented?**
   - **When presenting information do you prefer**
     1. Numbers
     2. Words
     3. Pictures
     4. Do you think a mixture is helpful
10. **Are there any other issues that you want to raise about options when you have a breech baby?**

## Topic guide for interviews with healthcare professionals

1. **Introduction of researcher and recap the purpose of the study.**
2. **Acknowledge that the interview/ focus group will be confidential**
3. **Do you have any questions or concerns about the study?**
4. **What information do you think should be included in a decision aid for women with a breech baby considering ECV?**
5. **What do you think is important to women when making a decision about breech presentation?**
6. **How do you think information should be presented to women?**
7. **How useful do you think information is when given as**

- **leaflets**
- **as a computer programme**
- **on the internet**
- **As a DVD**

1. **When presenting information which methods do you think are most useful?**

- **Numbers**
- **Words**
- **Pictures**
- **Do you think a mixture is helpful**

1. **What do you think are the advantages and disadvantages of the existing decision aid/**
   - **Do you think it could be adapted for use locally and if so, how do you think it could be adapted?**
2. **Are there any other issues that you want to raise about designing a decision aid for women considering ECV?**
